# Risk of Serious Falls Between Hemodialysis and Peritoneal Dialysis Patients: A Nationwide Population-based Cohort Study

**DOI:** 10.1038/s41598-020-64698-7

**Published:** 2020-05-08

**Authors:** Hsi-Hao Wang, Jia-Ling Wu, Yi-Che Lee, Li-Chun Ho, Min-Yu Chang, Hung-Hsiang Liou, Shih-Yuan Hung

**Affiliations:** 1 Division of Nephrology, Department of Internal medicine, E-DA Hospital, Kaohsiung, Taiwan; 2 School of Medicine, I-Shou University, Kaohsiung, Taiwan; 3 Department of Medical Quality, E-DA Hospital, Kaohsiung, Taiwan; 4 Department of Internal Medicine, National Cheng Kung University Hospital, College of Medicine, National Cheng Kung University, Tainan, Taiwan; 5 Division of Nephrology, Department of Internal medicine, E-DA Dachang Hospital, Kaohsiung, Taiwan; 6 Division of General Medicine, Department of Internal medicine, E-DA Hospital, Kaohsiung, Taiwan; 7 Division of Nephrology, Department of Internal Medicine, Hsin-Jen Hospital, New Taipei City, Taiwan

**Keywords:** Medical research, Nephrology, Risk factors

## Abstract

The association between serious falls and dialysis modality [hemodialysis (HD) and peritoneal dialysis (PD)] is unclear. A nationwide population-based retrospective cohort study with 127,823 end-stage renal disease patients aged over 18 years was conducted with the unmatched cohort of 101,304 HD and 7,584 PD patients retrieved from Taiwan’s National Health Insurance Research Database during 2000–2013. A total of 7,584 HD and 7,584 PD patients matched at 1:1 ratio by propensity score were enrolled to the study. Serious falls were defined by the diagnostic codes, E code, and image studies. Cox regression model and competing-risk model were used for statistical analysis. HD patients were older and had more comorbidities at baseline than PD patients. After matching and adjustment, HD patients had a higher risk of serious falls than PD patients [sHR 1.27 (95% CI 1.06–1.52)]. Females, elders, a history of falls before dialysis, comorbidity with stroke or visual problems, using diuretics, α-blockers, and mydriatics were associated with higher risks of serious falls among dialysis patients. The risk of serious falls was higher in HD patients than PD patients. Health professionals should create age-friendly environments, reduce unnecessary medications, and raise patients’ awareness of falls in daily life.

## Introduction

Dialysis patients have a higher risk for falls than general population^1–4^, with data suggesting fall rates of 1.2–1.7 per patient-year among patients treated with hemodialysis (HD) and peritoneal dialysis (PD)^5^. Dialysis patients with serious fall injuries such as hip fracture, joint dislocation, or brain injury are at greater risk for functional decline, loss of independence, poor quality of life, restrictions in mobility and social participation, nursing home placement, and high cost to the health system^6,7^. Most importantly, serious falls can result in hospitalization, disability, and mortality^1,7–9^.

Previous studies have shown that the risk factors for falls in dialysis patients include old age^1,5,7,10^, female^10^, malnutrition^1^, depression^1,10^, cognitive dysfunction^1^, a history of falls or stroke^1,11^, low Body Mass Index (BMI)^10^, frailty^8,12^, post-dialysis fatigue^7^, high comorbidity^5,10^, psychotropic drug use and polypharmacy^1,13,14^ as well as instability (hemodynamic and metabolic) caused by the dialysis therapy^2,7,15,16^. Some studies indicated that the risk of fall and hip fracture is higher in HD than in PD population^17–19^, but some reported that the fall rates were similar between both groups^5,20^. However, we still cannot conclude that which dialysis modality may have greater impact on the increasing fall risk among dialysis patients.

We designed this nationwide population-based retrospective cohort study to estimate the risk of serious falls among HD and PD patients to determine which dialysis modality would be more associated with serious falls.

## Methods

### Data source

The present study obtained data from Taiwan’s National Health Insurance Research Database (NHIRD), one of the largest and most comprehensive nationwide population reimbursement databases in the world^21^. This database contained electronic medical records of all patients insured by the National Health Insurance (NHI) program in Taiwan, with approximately 99.9% of Taiwan’s population (23 million) enrolled in this program by the end of 2014^22^. Taiwan government provided de-identified secondary datasets for scientists for research purposes. All dialysis patients in Taiwan are registered in this database, and we have retrieved related data from the subsets named “Registry for Catastrophic Illness Patients^23^.” This study was approved by the Institutional Review Board (IRB) of E-DA Hospital, Kaohsiung, Taiwan (EMRP-103-012).

### Study participants

**Figure 1:**
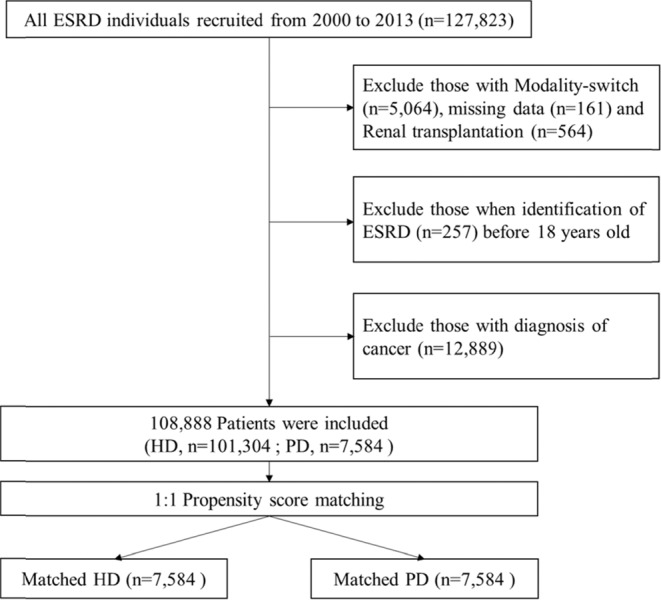
The flow chart for the establishment of matched-pairs of dialysis patients with either hemodialysis or peritoneal dialysis.

We selected all end-stage renal disease (ESRD) individuals who were older than 18 years and underwent dialysis from 1 January 2000 to 31 December 2013. ESRD was defined by the diagnostic codes of International Classification of Disease, Ninth Revision, Clinical Modification (ICD-9-CM = 585.X). A total of 127,823 incident ESRD patients comprised the unmatched cohort of 101,304 HD patients and 7,584 PD patients between 1 January 2000 and 31 December 2013 (Fig. 1). The index date of dialysis was defined as the first day after starting regular dialysis for 3 months. Two cohorts (HD and PD) were categorized from the ESRD patients. The exclusion criteria were patients under 18 years old when identification of ESRD, patients with modality-switch or renal transplantation, and patients diagnosed with any cancer. The endpoint of follow-up was the date of serious fall, death, or the end of 2013.

### Serious falls

The primary outcomes were serious falls that led dialysis patients to emergency department or to be hospitalized. Serious falls were defined by ICD-9-CM codes (800–904, 910–957) and an E code for a fall (E880-E889)^24^ combined with correlated image studies (X-ray and/or computer tomography). All study subjects were followed to ascertain whether they had subsequent hospitalization because of serious falls using outpatient (emergency) and inpatient claims from Taiwan’s NHIRD.

### Comorbidities

The baseline comorbidities had to be registered before the index day and were defined by ICD-9-CM codes, including coronary artery disease (410–413, 414.01–414.05, 414.8, 414.9), congestive heart failure (428, 398.91, 402.x1), stroke (430–438), hyperlipidemia (272), atrial fibrillation (427.31), hypertension (401–405), diabetes (250), dementia (290.0, 290.1, 290.2, 290.3, 290.4, 294.1, 331.0), osteoporosis (733.00), osteoarthritis (715.xx), liver cirrhosis (571.xx), and visual problems (362.02 diabetes mellitus with proliferative diabetic retinopathy (PDR), 368.9 visual disturbance, and 369.9 visual loss). Each comorbidity from outpatient claims needed to be confirmed again within the subsequent 12-month follow-up, and the first and last outpatient visits within 1 year had to be more than 30 days apart to prevent miscoding^25^.

### Medications

Previous studies indicated that polypharmacy^13^, central nervous system drugs, and psychotropics^14^ were associated with an increased risk of falls. Therefore, we also estimated the risk of serious falls when dialysis patients used different types of medications. The history of medications use was defined as medicines prescribed within 3 months before serious falls happened or within 3 months before the endpoint for those who didn’t fall.

### Statistical analyses

For a better comparability between HD and PD patients, we used propensity score matching on age, gender, index year of initiating dialysis, and initial comorbidities with a 1:1 ratio. Baseline characteristics and comorbidities for two cohorts were analyzed before and after matching. Differences between cohorts were evaluated using t-test for continuous variables and Chi-square test for categorical variables. The incidence of serious fall was calculated for two groups. The estimation of incidence rate was calculated under the Poisson assumption and the difference between HD and PD patients was compared by using the Poisson regression. The impact of different dialysis modalities on the risk of serious falls was analyzed by multivariable Cox proportional hazard model. The hazard ratios (HRs) with corresponding 95% confidence intervals (CI) were calculated and the proportional hazard assumption of the Cox models was assessed by a graphical method. To accurately assess fall risks between HD and PD population, death is an important competing risk factor. Thus, Fine and Gray competing-risk regression model is applied to estimate sub-distribution hazard ratio (sHR).

All statistical analyses were performed by using SAS statistical software (SAS System for Windows, Version 9.4, SAS Institute Inc., Cary, NC, USA). Results with two-sided P values less than 0.05 were considered statistically significant.

## Results

Before matching, mean ages of HD group and PD group were 63.42 and 54.52 years respectively, indicating that HD patients were generally older than PD patients. There was a higher proportion of males in the HD group (50.89%) than in the PD group (48.18%). HD patients (19.26%) were more likely to experience a history of falls before dialysis than PD patients (11.7%). HD patients (3.88 years) had longer duration of follow-up than PD patients (3.06 years). In addition, HD patients had higher prevalence rates of comorbidities than PD patients. After matching by propensity score, 7,584 HD and 7,584 PD patients were enrolled to the study. The baseline was almost balanced between two groups (Table 1) tested by standardized difference.

**Table 1:** Comparison of demographic and clinical characteristics of hemodialysis and peritoneal dialysis patients before and after matching by propensity score.

After matching	Before matching				After matching			
	All HD patients	All PD patients	P value	di	Matched HD patients	Matched PD patients	P value	di
Number of patients	101,304	7,584			7,584	7,584		
Age, No (%)								
Mean (SD)	63.42 (13.84)	54.52 (15.31)	<0.001	60.93	54.35 (15.13)	54.52 (15.31)	0.49	1.12
18–34 years	2,925 (2.89)	824 (10.86)	<0.001		839 (11.06)	824 (10.86)	0.46	
35–49 years	13,633 (13.46)	1,932 (25.47)			1,932 (25.47)	1,932 (25.47)		
50–64 years	33,971(33.53)	2,861(37.72)			2,869 (37.83)	2,861 (37.72)		
65–79 years	38,660 (38.16)	1,536 (20.25)			1,564 (20.62)	1,536 (20.25)		
≥80 years	12,115 (11.96)	431 (5.68)			380 (5.01)	431 (5.68)		
Sex (No of male, %)	51,556 (50.89)	3,654 (48.18)	<0.001	5.43	3,706 (48.87)	3,654 (48.18)	0.40	1.37
Index year			<0.001	68.62			0.86	1.18
2000–2001	11,982 (11.83)	109 (1.44)			120 (1.58)	109 (1.44)		
2002–2005	26,076 (25.74)	438 (5.78)			448 (5.91)	438 (5.78)		
2006–2009	31,506 (31.1)	3,314 (43.7)			3,321 (43.79)	3,314 (43.7)		
2010–2013	31,740 (31.33)	3,723 (49.09)			3,695 (48.72)	3,723 (49.09)		
Fall history	19,513 (19.26)	887 (11.70)	<0.001	21.03	1,125 (14.83)	887 (11.7)	<0.001	9.26
Duration of follow-up (years)	3.88 (3.31)	3.06 (2.31)	<0.001	28.73	3.49 (2.61)	3.06 (2.31)	<0.001	17.45
Baseline Comorbidities (%)								
Coronary artery disease	35,239 (34.79)	1,725 (22.75)	<0.001	26.84	1,684 (22.2)	1,725 (22.75)	0.43	1.30
Congestive heart failure	37,546 (37.06)	1,704 (22.47)	<0.001	32.33	1,685 (22.22)	1704 (22.47)	0.71	0.60
Stroke	27,098 (26.75)	1,109 (14.62)	<0.001	30.28	1,091 (14.39)	1,109 (14.62)	0.68	0.67
Hyperlipidemia	44,556 (43.98)	3,372 (44.46)	0.42	0.97	3,347 (44.13)	3,372 (44.46)	0.68	0.66
Atrial fibrillation	4,593 (4.53)	210 (2.77)	<0.001	9.42	143 (1.89)	210 (2.77)	<0.001	5.86
Hypertension	88,127 (86.99)	6,223 (82.05)	<0.001	13.69	6,243 (82.32)	6,223 (82.05)	0.67	0.69
Diabetes mellitus	44,393 (43.82)	2,498 (32.94)	<0.001	22.52	2,368 (31.22)	2,498 (32.94)	0.02	3.67
Dementia	3,942 (3.89)	150 (1.98)	0.01	11.36	139 (1.83)	150 (1.98)	0.51	1.06
Osteoporosis	5,743 (5.67)	301 (3.97)	0.04	7.94	255 (3.36)	301 (3.97)	0.05	3.23
Osteoarthritis	22,579 (22.29)	1,090 (14.37)	0.03	20.57	1,039 (13.7)	1,090 (14.37)	0.23	1.94
Liver cirrhosis	5,184 (5.12)	217 (2.86)	<0.001	11.55	172 (2.27)	217 (2.86)	0.02	3.75
Visual problems*	19,181 (18.93)	1,021 (13.46)	<0.001	14.89	1,152 (15.19)	1,021 (13.46)	0.0024	4.93

Note: di, standardized differences with absolute values> 10% considered as residual imbalance.

*Visual problems including proliferative diabetic retinopathy, visual disturbance, visual loss.

Before matching, HD patients had higher crude incidence rate of serious falls than PD patients (15.97 (15.58–16.37) versus 8.79 (7.63–10.08) per 1,000 patient-years, P < 0.001). Females and the elders were prone to serious falls in both HD and PD groups. Moreover, in both HD and PD groups the incidence of serious falls increased with age for both males and females. After matching, incidence of serious falls was similar between two groups (9.22 (8.10–10.46) versus 8.79 (7.63–10.08) per 1,000 patient-years, P = 0.61). Before matching, HD population had higher mortality rate than PD population (120.48 (119.40–121.57) versus 93.73 (89.83–97.75) per 1,000 patient-years, P < 0.001) (Table 2). However, the mortality rate was lower in the HD population than in the PD population (74.21 (70.96–77.56) versus 93.73 (89.83–97.75) per 1,000 patient-years, P < 0.001) after matching. PD patients had equal survival rate only in the first year and then had a higher mortality risk in the follow-up years (see supplementary Table 2).

**Table 2:** Comparison of incidence rates (per 1,000 patient-years) of serious fall between hemodialysis and peritoneal dialysis patients before and after matching by propensity score.

Characteristics	Before matching				P value	After matching				P value
	All HD patients		All PD patients			Matched HD patients		Matched PD patients		
	No. of events	Incidence rates	No. of events	Incidence rates		No. of events	Incidence rates	No. of events	Incidence rates	
Overall	6,286	15.97 (15.58–16.37)	204	8.79 (7.63–10.08)	<0.001	244	9.22 (8.10–10.46)	204	8.79 (7.63–10.08)	0.61
Male	2,500	12.89 (12.39–13.41)	73	6.97 (5.46–8.76)	<0.001	97	7.86 (6.38–9.59)	73	6.97 (5.46–8.76)	0.43
Age (years)										
18–34 years	46	4.66 (3.41–6.22)	3	2.42 (0.50–7.08)		5	3.15 (1.02–7.34)	3	2.42 (0.50–7.08)	
35–49 years	260	6.46 (5.70–7.30)	10	3.32 (1.59–6.11)		18	5.28 (3.13–8.34)	10	3.32 (1.59–6.11)	
50–64 years	772	10.05 (9.36–10.79)	28	6.36 (4.23–9.19)		40	8.12 (5.8–11.05)	28	6.36 (4.23–9.19)	
65–79 years	1,091	19.32 (18.19–20.50)	25	15.91 (10.29–23.48)		22	10.49 (6.57–15.88)	25	15.91 (10.29–23.48)	
≥80 years	331	31.40 (28.11–34.98)	7	27.18 (10.93–56.00)		12	38.49 (19.89–67.24)	7	27.18 (10.93–56)	
Female	3,786	18.97 (18.37–19.58)	131	10.29 (8.61–12.22)	<0.001	147	10.41 (8.8–12.24)	131	10.29 (8.61–12.22)	0.92
Age (years)										
18–34 years	26	3.51 (2.29–5.14)	6	3.44 (1.26–7.48)		8	4.42 (1.91–8.70)	6	3.44 (1.26–7.48)	
35–49 years	209	5.53 (4.80–6.33)	12	3.09 (1.60–5.40)		18	4.26 (2.53–6.74)	12	3.09 (1.6–5.40)	
50–64 years	1,062	14.91 (14.03–15.83)	37	7.80 (5.49–10.75)		49	9.49 (7.02–12.54)	37	7.80 (5.49–10.75)	
65–79 years	1,998	28.51 (27.27–29.79)	58	28.96 (21.99–37.44)		57	23.32 (17.66–30.21)	58	28.96 (21.99–37.44)	
≥80 years	491	37.58 (34.33–41.05)	18	50.96 (30.20–80.53)		15	31.57 (17.67–52.08)	18	50.96 (30.2–80.53)	
Mortality	47,412	120.48 (119.40–121.57)	2,175	93.73 (89.83–97.75)	<0.001	1,963	74.21(70.96–77.56)	2,175	93.73 (89.83–97.75)	<0.001

Table 3 showed the results of relative risk of incidence of serious falls between HD and PD patients. With adjustment for confounders, there is no obvious increased risk of serious falls in HD patients compared with PD patients by Cox model [HR 1.11 (95% CI = 0.93–1.33)]. However, HD patients had a higher risk of serious falls than PD patients in the Fine and Gray model, showing sHR 1.27 (95% CI = 1.06–1.52). Males had a HR of 0.69 (95% CI = 0.58–0.84) and sHR of 0.67 (95% CI = 0.55–0.81) for serious falls, indicating that women had a significantly higher risk of serious falls than men. The elders had a HR of 1.05 (95% CI = 1.04–1.06) and sHR of 1.03 (95% CI = 1.02–1.04) for serious falls compared with younger patients. People had a history of falls before dialysis showed a significantly increased risk of serious falls than those who never fall, with HR of 1.61 (95% CI = 1.31–1.98) and sHR of 1.47 (95% CI = 1.19–1.82). Patients who have experienced stroke had a higher risk of serious falls than patients with no history of stroke, with HR of 1.42 (95% CI = 1.14–1.76) and sHR of 1.25 (95% CI = 1.00–1.56). Patients with visual problems had 50% increased risk of serious falls than those with normal visual acuity, showing HR of 1.52 (95% CI = 1.18–1.94) and sHR of 1.50 (95% CI = 1.16–1.93). Patients who used α-blocker agents during dialysis had an almost 30% higher risk of serious falls compared to those who did not, showing HR of 1.29 (95% CI = 1.02–1.65) and sHR of 1.28 (95% CI = 1.00–1.63). Patients who used diuretics during dialysis had a significantly higher risk of serious falls compared to those who did not, showing HR of 1.82 (95% CI = 1.49–2.23) and sHR of 1.26 (95% CI = 1.03–1.54). Patients who used mydriatics had a 2–3 times higher risk of serious falls than those who did not use, showing HR of 2.61 (95% CI = 1.28–5.30) and sHR of 2.97 (95% CI = 1.40–6.27).

**Table 3:** Analysis of relative risk of incidence of fall between patients receiving hemodialysis and peritoneal dialysis after matching by multivariable Cox proportional hazard model and multivariable subdistribution hazard models.

Variable	Cox proportional hazard model	Competing Risk Regression model
	HR (95% CI)	HR (95% CI)
Dialysis modality (Reference = PD)	1.11 (0.93–1.33)	1.27 (1.06–1.52)
Sex (Reference = female)	0.69 (0.58–0.84)	0.67 (0.55–0.81)
Age	1.05 (1.04–1.06)	1.03 (1.02–1.04)
Fall history	1.61 (1.31–1.98)	1.47 (1.19–1.82)
Comorbidities		
Coronary artery disease	0.99 (0.81–1.22)	1.00 (0.81–1.24)
Congestive heart failure	1.21 (0.98–1.49)	1.06 (0.86–1.32)
Stroke	1.42 (1.14–1.76)	1.25 (1.00–1.56)
Hyperlipidemia	0.99 (0.82–1.20)	0.99 (0.82–1.20)
Atrial fibrillation	0.68 (0.39–1.19)	0.61 (0.35–1.08)
Hypertension	1.04 (0.78–1.38)	1.11 (0.84–1.48)
Diabetes mellitus	1.13 (0.91–1.40)	0.95 (0.75–1.19)
Dementia	1.33 (0.84–2.11)	0.99 (0.62–1.58)
Osteoporosis	0.95 (0.67–1.34)	0.94 (0.66–1.35)
Osteoarthritis	1.10 (0.88–1.36)	1.12 (0.90–1.40)
Liver cirrhosis	0.9 (0.51–1.56)	0.75 (0.43–1.31)
Visual problems	1.52 (1.18–1.94)	1.50 (1.16–1.93)
Medication		
Narcotics	1.07 (0.85–1.34)	0.82 (0.64–1.04)
α-blocker agents	1.29 (1.02–1.65)	1.28 (1.00–1.63)
Diuretics	1.82 (1.49–2.23)	1.26 (1.03–1.54)
Anticonvulsants	1.41 (0.94–2.10)	1.42 (0.96–2.12)
Skeletal Muscle Relaxants	1.60 (0.90–2.87)	1.40 (0.79–2.49)
Parkinsonian Agents	0.84 (0.51–1.39)	0.89 (0.53–1.49)
Sedatives and Hypnotics	0.98 (0.81–1.19)	0.98 (0.81–1.19)
Anxiety Anxiolytics	0.95 (0.39–2.33)	0.98 (0.40–2.43)
Antipsychotic	0.77 (0.51–1.17)	0.76 (0.49–1.16)
Antidepression	1.15 (0.86–1.53)	1.13 (0.84–1.51)
Antihistamines	1.17 (0.94–1.45)	1.21 (0.97–1.50)
Mydriatic agent	2.61 (1.28–5.30)	2.97 (1.40–6.27)

## Discussion

Dialysis patients experience a greater fall risk compared to the general population^1,4,7–9^. However, it is still unclear regarding different dialysis modalities (HD and PD) and the consequence of falls risk in ESRD patients.

Our finding suggested that HD patients had worse baseline characteristics than PD patients before matching, including older age, more cardiovascular diseases (CVD) and comorbidities, and a history of more falls before dialysis; these baseline characteristics may lead to the consequences of higher crude incidence of serious falls and higher crude mortality rate. However, the incidence of serious falls was similar between the two groups after matching. Furthermore, although there is no obvious increased risk of serious falls in HD patients compared with PD patients after adjustment in the Cox model, HD patients had a higher risk of serious falls than PD patients in the Fine and Gray model after considering competing risk.

Our finding is consistent with several studies that suggested the risk of hip fracture is higher in the HD population than in the PD population. As over 90% of hip fracture are the result of a fall^26^, it may infer that the fall risk is higher in HD patients than PD patients. Besides, the intermittent nature of HD may lead to fluctuation of fluid status, hemodynamic instability, and orthostatic hypotension. For example, Mathew AT *et al*. analyzed 842,028 HD and 87,086 PD patients between 1992 and 2009, and concluded that HD patients had 1.6 times greater odds of hip fracture than PD patients after adjustment for risk factors^18^. A population-based cohort study including 28,048 HD and 3,506 PD patients from 1998 to 2008 reported the incidence of hip fracture was 13.60 per 1000 patient-years in the HD group and 6.25 in the PD group. In addition, HD had a greater hip fracture risk compared to PD^17^. Lin ZZ *et al*. also used NHIRD to analyze 51,473 patients who started dialysis between 1999 and 2005 and found that HD patients had a 31% higher incidence of hip fracture than PD patients^19^.

This result is in contrast to previous studies. Farragher J. conducted a prospective cohort study to compare the falls risk between cohorts of 236 elderly patients maintained on HD and PD, and concluded that accidental falls are equally common in the PD and HD patients^20^. Farragher disproved the hypothesis that PD patients have lower fall rates than HD patients. This might be the result of PD patients encountering more inconveniences in daily life such as connecting tube around the bed at night, and decreased well-being and worse health^20^. European researchers undertook a study following 114 patients to assess the prevalence of falls and the impact on mortality and quality of life in elderly patients on HD and PD. They also concluded that fall incidence is comparable between HD and PD^27^.

Our study consistently supported that women, the elderly, and a history of falls before dialysis and stroke were associated with significantly higher risks of serious falls among dialysis patients as proved in previous studies. It is possible that physical, sensory, and cognitive changes connected with aging and/or stroke resulted in the higher falls risk in older dialysis population. The fact that women showed a greater falls risk compared to men is probably related to less physical activity, lower bone mass, weaker muscles and lower body strength in women than in men^28^. Patients with falls history before dialysis had a higher risk of recurrent falls than non-fallers, which was consistent with previous studies. One US study concluded that patients with a serious fall had 2.7-fold higher rates of serious fall injuries in the first year after the start of dialysis^11^. Another Canadian study reported that a history of one or more falls in the previous year was an independent risk factor for falls^5^.

In this analysis, we found that diuretics, α-blockers, and mydriatics were the possible risk factors for serious falls among dialysis patients. This finding had not been commonly mentioned in previous documents. In the present study, dialysis patients prescribed with diuretics showed an approximately 40–95% significantly higher risk of serious falls compared to those who did not use diuretics. Diuretics and α-blockers have been classified as “fall-risk-increasing drugs” (FRIDs) because some of the side effects such as frequent urination, fatigue, dizziness, lightheadedness, muscle cramps, and postural hypotension might make people fall down,^29–32^. Our study also found that mydriatics were associated with a 2–3 times significantly higher risk of serious falls among dialysis patients compared to those who did not use mydriatics. Mydriatics were used to paralyze and relax the iris muscle and to dilate the pupil in the eye for diagnostic procedures. Common side effects of mydriatics include blurred vision, decreased vision, and eye sensitivity to light, which could be the possible explanations for a high risk of falls in the dialysis population^32^. In addition, patients who need to use mydriatics implied that they had visual problems or eye diseases that need ophthalmic exams. According to previous studies, loss of visual function was associated with a 2 times higher risk for falls or fall-related injuries in elderly patients^33–35^. Jack CI *et al*. found that there was a particularly high prevalence in elderly patients who were admitted with falls^36^. In this study we also found that patients with visual problems did have higher risks for serious falls. Postural balance changes due to impaired vestibular, neurological and musculoskeletal functions were shown in hemodialysis and peritoneal patients^2,16^. Vision is an important compensation in maintaining balance physiologically, and our data suggested mydriatics or impaired vision was a significant risk factor for serious falls among PD and HD patients.

**Figure 2:**
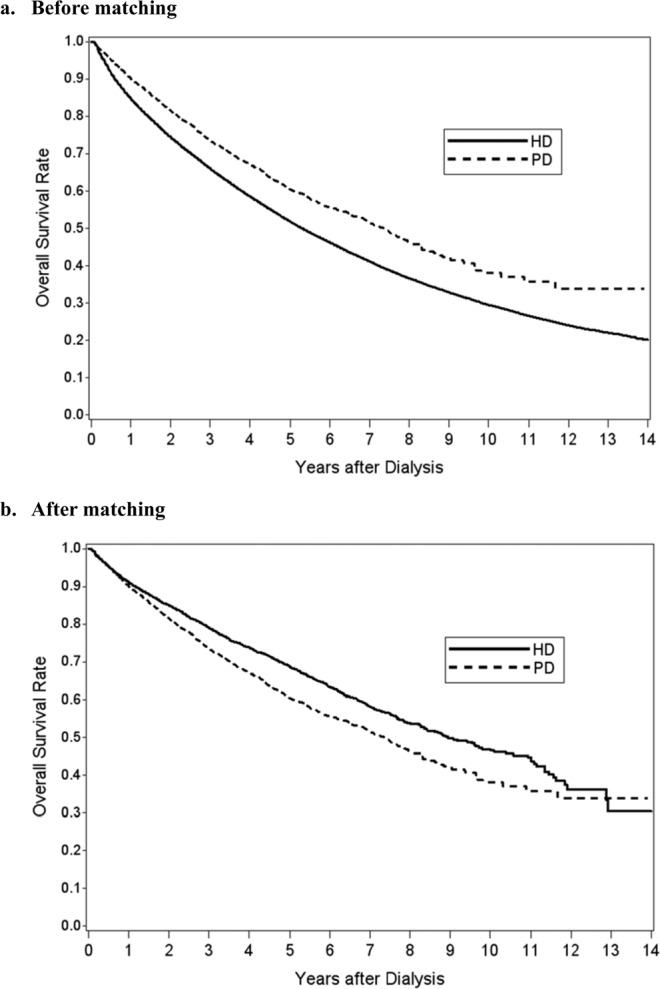
The 14 years survival rates were subsequently estimated by the Kaplan-Meier method between patients receiving hemodialysis and peritoneal dialysis before and after matching.

Although we found that HD patients had a higher mortality rate than PD patients at baseline, the mortality rate became lower in HD group compared to PD group after matching. This finding is consistent with one meta-analysis that suggested a higher risk of death in elderly patients receiving PD than in those receiving HD^37^. Another study stated that PD patients had a lower risk of death than HD patients in the first 3 months of dialysis and the survival advantage would last for 1.5–2 years. Nevertheless, the mortality risk would change over time and become equal or greater in PD compared to HD^38^. Our finding is inconsistent with a retrospective study in Canada, which concluded that HD and PD are associated with similar mortality and the effect of modality on survival does not appear to change over time^39^. However, our study represented that PD patients had better performance of survival curve than HD patients only before matching (Fig. 2). Higher rates of technique failure, fluid overload due to poor adherence, low effluent drain volume, high transporter status, higher protein loss during dialysis process, metabolic derangement caused by glucose-based dialysate, and PD related infection may lead to shorter survival and inferior outcome in PD patients^40–43^.

This study has several strengths. First, with population-based cohort, we conquered the weakness of small sample size in previous studies. Second, there is limited research on medication and associated risk of falls among dialysis patients; however, our study estimated the risks of different medications for serious falls among dialysis patients. Third, we used propensity score matching to minimize the selection bias. Fourth, we used Cox model to control potential confounders and investigate the impact of different dialysis modalities on the risk of serious falls. Finally, we considered death and serious falls as competing risks so the Fine and Gray model were applied to calculate the sHR as validation of our finding.

There are several limitations in our study. First, selection bias is an inherent limitation in the nature of retrospective study. To compensate for this disadvantage, we used full data retrieved from the NHI program with the entire dialysis population extending across a 14-year period in Taiwan, and the sample size was large enough to permit propensity matching and minimize selection bias. Second, some important variables that might affect the risk of serious falls, such as frailty, post-dialysis fatigue, malnutrition, and gait speed were not available in the NHIRD. However, these factors were usually related to dialysis modality and comorbid conditions which were controlled in our model construction, and they did not seem to contribute much differential bias in this study. Third, not only intrinsic but also extrinsic risk factors were associated with fall accidents. As most population-based studies, we had no information about environmental hazards, bad quality footwear, inappropriate walking aids, and caregiver factors from each patient in our claims data. Further prospective study is needed to take these considerations into account. Forth, patients who didn’t seek for medical help would not have a record in the NHIRD. Consequently, serious falls that led to sudden deaths in patients’ homes were not included in this study. However, Taiwan is the 17th densely populated country in the world and has a compulsory national health insurance program with a 99.9% coverage rate. Should an emergency occur, people who need help will be easily found and get medical services soon. Therefore, the true effects of these data omissions will be minimal and equal in both hemodialysis and peritoneal dialysis patients. Finally, statistical matching cannot postulate the general wellbeing. As we all know, PD patients are generally more active, independent, educated, and less comorbid people compared to HD patients. As a result, this study found that less comorbid and younger HD patients have better survival and a similar risk of falls with all PD patients estimated by Cox regression model. In this regard, the dialysis modality does not affect the fall rates of HD patients. Nevertheless it is not always possible to apply this result to all ESRD patients, including the majority of HD patients with high comorbidity burden. We should be very cautious while extending the results to all ESRD patients in our daily practice.

In conclusion, the risk of serious falls was higher in HD patients than PD patients after adjustment by Fine and Gray competing-risk regression model. However, the mortality rate was lower in HD patients than PD patients after matching. Females, elders, a history of fall before dialysis, comorbidity with stroke or visual problems, using diuretics, α-blockers, and mydriatics were the possible risk factors for serious falls among dialysis patients. Health professionals should make more efforts to create age-friendly environments, reduce unnecessary medications, and raise patients’ awareness of falls in daily life.

## Supplementary information

Supplementary Information.

## Data Availability

Taiwan’s National Health Insurance Research Database (NHIRD) was provided by the National Health Insurance Administration, Ministry of Health and Welfare, Taiwan. Relevant information and applications are available at the following website https://nhird.nhri.org.tw/en/index.html

## References

[1] Rossier A, Pruijm M, Hannane D, Burnier M, Teta D (2012). Incidence, complications and risk factors for severe falls in patients on maintenance haemodialysis. Nephrology, dialysis, transplantation: official publication of the European Dialysis and Transplant Association - European Renal Association.

[2] Erken E (2016). The effect of hemodialysis on balance measurements and risk of fall. International urology and nephrology.

[3] Toussaint ND, Elder GJ, Kerr PG (2010). A rational guide to reducing fracture risk in dialysis patients. Seminars in dialysis.

[4] Cook WL (2006). Falls and fall-related injuries in older dialysis patients. Clinical journal of the American Society of Nephrology: CJASN.

[5] Farragher J (2014). Accidental falls and risk of mortality among older adults on chronic peritoneal dialysis. Clinical journal of the American Society of Nephrology: CJASN.

[6] Plantinga LC, Patzer RE, Franch HA, Bowling CB (2017). Serious Fall Injuries Before and After Initiation of Hemodialysis Among Older ESRD Patients in the United States: A Retrospective Cohort Study. American journal of kidney diseases: the official journal of the National Kidney Foundation.

[7] Abdel-Rahman EM, Turgut F, Turkmen K, Balogun RA (2011). Falls in elderly hemodialysis patients. QJM: An International Journal of Medicine.

[8] McAdams-DeMarco MA (2013). Frailty and falls among adult patients undergoing chronic hemodialysis: a prospective cohort study. BMC nephrology.

[9] Li M, Tomlinson G, Naglie G, Cook WL, Jassal SV (2008). Geriatric comorbidities, such as falls, confer an independent mortality risk to elderly dialysis patients. Nephrology, dialysis, transplantation: official publication of the European Dialysis and Transplant Association - European Renal Association.

[10] Bowling CB (2016). Association of Reduced eGFR and Albuminuria with Serious Fall Injuries among Older Adults. Clinical journal of the American Society of Nephrology: CJASN.

[11] Bowling CB, Hall RK, Khakharia A, Franch HA, Plantinga LC (2018). Serious Fall Injury History and Adverse Health Outcomes After Initiating Hemodialysis Among Older U.S. Adults. The journals of gerontology. Series A, Biological sciences and medical sciences.

[12] Delgado C (2015). Association of Self-Reported Frailty with Falls and Fractures among Patients New to Dialysis. American journal of nephrology.

[13] Guideline for the prevention of falls in older persons (2001). American Geriatrics Society, British Geriatrics Society, and American Academy of Orthopaedic Surgeons Panel on Falls Prevention. Journal of the American Geriatrics Society.

[14] Hartikainen S, Lonnroos E, Louhivuori K (2007). Medication as a risk factor for falls: critical systematic review. The journals of gerontology. Series A, Biological sciences and medical sciences.

[15] Wang IK (2015). Comparison of Subdural Hematoma Risk between Hemodialysis and Peritoneal Dialysis Patients with ESRD. Clinical journal of the American Society of Nephrology: CJASN.

[16] Analan PD, Ozelsancak R (2019). Balance and fall risk in peritoneal dialysis patients. Journal of back and musculoskeletal rehabilitation.

[17] Chen YJ (2014). Greater risk of hip fracture in hemodialysis than in peritoneal dialysis. Osteoporosis international: a journal established as result of cooperation between the European Foundation for Osteoporosis and the National Osteoporosis Foundation of the USA.

[18] Mathew AT (2014). Increasing hip fractures in patients receiving hemodialysis and peritoneal dialysis. American journal of nephrology.

[19] Lin ZZ (2014). Epidemiology and mortality of hip fracture among patients on dialysis: Taiwan National Cohort Study. Bone.

[20] Farragher J (2016). Equivalent Fall Risk in Elderly Patients on Hemodialysis and Peritoneal Dialysis. Peritoneal dialysis international: journal of the International Society for Peritoneal Dialysis.

[21] Chang CC, Liao CC, Chen TL (2016). Perioperative medicine and Taiwan National Health Insurance Research Database. Acta anaesthesiologica Taiwanica: official journal of the Taiwan Society of Anesthesiologists.

[22] National Health Insurance Administration & Ministry of Health and Welfare. (Taiwan, R.O.C., 2014).

[23] National Health Insurance Administration & Taiwan. Catastrophic Illness Record, <https://www.nhi.gov.tw/english/Content_List.aspx?n=E6424A1BD94E913B&topn=BCB2B0D2433F6491> (2019).

[24] Tinetti, M. E. *et al*. Antihypertensive medications and serious fall injuries in a nationally representative sample of older adults. *JAMA Intern Med*, **174**(4), 588–595. Retrieved from, https://www.ncbi.nlm.nih.gov/pubmed/24567036, 10.1001/jamainternmed.2013.1476 (2014). 10.1001/jamainternmed.2013.14764 PMC4136657 24567036

[25] Chen HF, Ho CA, Li CY (2006). Age and sex may significantly interact with diabetes on the risks of lower-extremity amputation and peripheral revascularization procedures: evidence from a cohort of a half-million diabetic patients. Diabetes care.

[26] Grisso JA (1991). Risk factors for falls as a cause of hip fracture in women. The Northeast Hip Fracture Study Group. The. New England journal of medicine.

[27] van Loon IN (2019). The prevalence and impact of falls in elderly dialysis patients: Frail elderly Patient Outcomes on Dialysis (FEPOD) study. Archives of gerontology and geriatrics.

[28] Stevens JA, Sogolow ED (2005). Gender differences for non-fatal unintentional fall related injuries among older adults. Injury prevention: journal of the International Society for Child and Adolescent. Injury Prevention.

[29] de Groot MH (2013). The effects of fall-risk-increasing drugs on postural control: a literature review. Drugs & aging.

[30] Cheung BM, Wong YL, Lau CP (2005). Queen Mary Utilization of Antihypertensive Drugs Study: side-effects of antihypertensive drugs. Journal of clinical pharmacy and therapeutics.

[31] Sica DA, Carter B, Cushman W, Hamm L (2011). Thiazide and loop diuretics. Journal of clinical hypertension (Greenwich, Conn.).

[32] Rengstorff RH, Doughty CB (1982). Mydriatic and cycloplegic drugs: a review of ocular and systemic complications. American journal of optometry and physiological optics.

[33] Felson DT (1989). Impaired vision and hip fracture. The Framingham Study. Journal of the American Geriatrics Society.

[34] Gerson LW, Jarjoura D, McCord G (1989). Risk of imbalance in elderly people with impaired hearing or vision. Age and ageing.

[35] Salonen L, Kivela SL (2012). Eye diseases and impaired vision as possible risk factors for recurrent falls in the aged: a systematic review. Current gerontology and geriatrics research.

[36] Jack CI, Smith T, Neoh C, Lye M, McGalliard JN (1995). Prevalence of low vision in elderly patients admitted to an acute geriatric unit in Liverpool: elderly people who fall are more likely to have low vision. Gerontology.

[37] Han SS (2015). Dialysis Modality and Mortality in the Elderly: A Meta-Analysis. Clinical journal of the American Society of Nephrology: CJASN.

[38] Sinnakirouchenan R, Holley JL (2011). Peritoneal dialysis versus hemodialysis: risks, benefits, and access issues. Advances in chronic kidney disease.

[39] Wong B (2018). Comparison of Patient Survival Between Hemodialysis and Peritoneal Dialysis Among Patients Eligible for Both Modalities. American journal of kidney diseases: the official journal of the National Kidney Foundation.

[40] Brimble KS, Walker M, Margetts PJ, Kundhal KK, Rabbat CG (2006). Meta-analysis: peritoneal membrane transport, mortality, and technique failure in peritoneal dialysis. Journal of the American Society of Nephrology: JASN.

[41] Dalal P, Sangha H, Chaudhary K (2011). In Peritoneal Dialysis, Is There Sufficient Evidence to Make “PD First”. Therapy? International journal of nephrology.

[42] Griva K (2014). Non-adherence in patients on peritoneal dialysis: a systematic review. PloS one.

[43] Stegmayr, B. Dialysis Procedures Alter Metabolic Conditions. *Nutrients* **9**, 10.3390/nu9060548 (2017). 10.3390/nu9060548 PMC5490527 28554992

