# Supplementary material for: Risk of Serious Falls Between Hemodialysis and Peritoneal Dialysis Patients: A Nationwide Population-based Cohort Study
Source: Sci Rep. 2020 May 8;10:7799. doi: 10.1038/s41598-020-64698-7 (PMC7211016; doi:10.1038/s41598-020-64698-7)
Supplement: Supplementary file 1 — Supplementary Information. [file 41598_2020_64698_MOESM1_ESM.docx]

**Risk of Serious Falls Between Hemodialysis and Peritoneal Dialysis Patients: A Nationwide Population-based Cohort Study**

Hsi-Hao Wang, MD, MPH ^1, 2, 3^, Jia-Ling Wu, MSc ^4^, Yi-Che Lee, MD, PhD ^2,5^,

Li-Chun Ho, MD, PhD ^1,2,6^, Min-Yu Chang, MD, MS ^1,2^, Hung-Hsiang Liou, MD^7^, Shih-Yuan Hung*, MD ^1, 2^

1. Division of Nephrology, Department of Internal medicine, E-DA Hospital, Kaohsiung, Taiwan.

2. School of Medicine, I-Shou University, Kaohsiung, Taiwan

3. Department of Medical Quality, E-DA Hospital, Kaohsiung, Taiwan.

4. Department of Internal Medicine, National Cheng Kung University Hospital, College of Medicine, National Cheng Kung University, Tainan, Taiwan

5. Division of Nephrology, Department of Internal medicine, E-DA Dachang Hospital, Kaohsiung, Taiwan.

6. Division of General Medicine, Department of Internal medicine, E-DA Hospital, Kaohsiung, Taiwan.

7. Division of Nephrology, Department of Internal Medicine, Hsin-Jen Hospital, New Taipei City, Taiwan.

* **Correspondence:**

Shih-Yuan Hung

*Internal Medicine Department, Division of Nephrology, E-DA Hospital, Kaohsiung, Taiwan.*

Address: No.1, Yida Road, Jiaosu Village, Yanchao District, Kaohsiung City 82445, Taiwan, R.O.C.

Tel: +886-7-6150011#1732

Email: ed100367@edah.org.tw (SYH)

**Supplementary Table 1. Analysis of relative risk of all-cause mortality between patients receiving hemodialysis and peritoneal dialysis before and after matching by multivariable Cox proportional hazard model.**

| **Variable** | **Before matching** | **After matching** |
| --- | --- | --- |
|  | **HR (95% CI)** | **HR (95% CI)** |
| **Univariate analysis**  **Dialysis modality (Reference=PD)** | 1.37 (1.31-1.43) | 0.79 (0.75-0.84) |
| **Multivariable analysis**  **Dialysis modality (Reference=PD)** | 0.83 (0.80-0.87) | 0.75 (0.71-0.80) |
| **Sex (Reference=female)** | 1.18 (1.16-1.20) | 1.29 (1.22-1.38) |
| **Age** | 1.05 (1.04-1.05) | 1.04 (1.04-1.04) |
| **Comorbidities** |  |  |
| Coronary artery disease | 1.09 (1.07-1.11) | 1.13 (1.06-1.22) |
| Congestive heart failure | 1.32 (1.30-1.35) | 1.45 (1.35-1.55) |
| Stroke | 1.35 (1.33-1.38) | 1.40 (1.30-1.51) |
| Hyperlipidemia | 0.89 (0.87-0.90) | 0.98 (0.92-1.05) |
| Atrial fibrillation | 1.34 (1.29-1.39) | 1.27 (1.11-1.47) |
| Hypertension | 0.95 (0.92-0.98) | 0.90 (0.81-0.99) |
| Diabetes mellitus | 1.70 (1.67-1.73) | 1.89 (1.77-2.02) |
| Dementia | 1.46 (1.41-1.52) | 1.67 (1.44-1.94) |
| Osteoporosis | 1.03 (1.00-1.07) | 1.08 (0.95-1.23) |
| Osteoarthritis | 0.99 (0.97-1.02) | 1.04 (0.96-1.12) |
| Liver cirrhosis | 1.66 (1.61-1.72) | 1.88 (1.65-2.15) |

**Supplementary Table 2. Analysis of relative risk of all-cause mortality between patients receiving hemodialysis and peritoneal dialysis stratified by dialysis time before and after matching by multivariable Cox proportional hazard models.**

| **Variable** | **Before matching** | | **After matching** | |
| --- | --- | --- | --- | --- |
|  | **< 1 years** | **≧1 years** | **< 1 years** | **≧1 years** |
|  | **HR (95% CI)** | **HR (95% CI)** | **HR (95% CI)** | **HR (95% CI)** |
| **Dialysis modality (Reference=PD)** | 1.01 (0.93-1.09) | 0.75 (0.72-0.79) | 0.95 (0.85-1.06) | 0.68 (0.63-0.73) |
| **Sex (Reference=female)** | 1.12 (1.09-1.16) | 1.21 (1.18-1.23) | 1.18 (1.05-1.32) | 1.35 (1.25-1.45) |
| **Age** | 1.05 (1.04-1.05) | 1.05 (1.04-1.05) | 1.04 (1.04-1.05) | 1.04 (1.04-1.04) |
| **Comorbidities** |  |  |  |  |
| Coronary artery disease | 1.09 (1.05-1.13) | 1.09 (1.06-1.11) | 1.09 (0.97-1.24) | 1.16 (1.06-1.26) |
| Congestive heart failure | 1.37 (1.32-1.41) | 1.31 (1.28-1.34) | 1.60 (1.42-1.81) | 1.39 (1.27-1.51) |
| Stroke | 1.53 (1.48-1.58) | 1.28 (1.25-1.31) | 1.50 (1.32-1.70) | 1.36 (1.25-1.49) |
| Hyperlipidemia | 0.81 (0.78-0.84) | 0.92 (0.90-0.94) | 0.93 (0.82-1.04) | 1.00 (0.93-1.08) |
| Atrial fibrillation | 1.44 (1.37-1.53) | 1.26 (1.20-1.32) | 1.42 (1.14-1.77) | 1.18 (0.98-1.42) |
| Hypertension | 0.81 (0.76-0.85) | 1.01 (0.97-1.05) | 0.80 (0.67-0.95) | 0.95 (0.84-1.07) |
| Diabetes mellitus | 1.53 (1.48-1.58) | 1.78 (1.74-1.82) | 1.62 (1.44-1.82) | 2.03 (1.87-2.20) |
| Dementia | 1.49 (1.41-1.58) | 1.41 (1.34-1.48) | 1.68 (1.36-2.09) | 1.62 (1.32-2.00) |
| Osteoporosis | 1.03 (0.97-1.09) | 1.03 (0.99-1.07) | 1.05 (0.84-1.31) | 1.09 (0.94-1.28) |
| Osteoarthritis | 0.99 (0.96-1.03) | 1.00 (0.97-1.02) | 0.97 (0.85-1.12) | 1.07 (0.97-1.17) |
| Liver cirrhosis | 1.93 (1.83-2.04) | 1.53 (1.46-1.59) | 2.08 (1.68-2.59) | 1.78 (1.50-2.11) |
